# *A disintegrin and metallopeptidase domain (ADAM) 12*,* ADAM 17* mRNA and ADAM10 protein hold potential as biomarkers for detection of early gastric cancer

**DOI:** 10.1038/s41598-024-84237-y

**Published:** 2025-01-04

**Authors:** Sooyeon Oh, Sang-Soo Lee, Hoeyoung Jin, Seo-Hyeon Choi, Choong-Keun Cha, Jooho Lee, KyuBum Kwack, Sang Gyun Kim, Sang-Woon Choi

**Affiliations:** 1 Chaum Life Center, CHA University School of Medicine, Seoul, 06062 Korea; 2 Graduate school of Internal Medicine, Seoul National University College of Medicine, Seoul, 03080 Korea; 3 Department of Biomedical Science, College of Life Science, CHA University, Seongnam, 13488 Korea; 4 Department of Gastroenterology and Hepatology, CHA Bundang Medical Center, CHA University School of Medicine, Seongnam, 13496 Korea; 5 Division of Gastroenterology, Department of Internal Medicine and Liver Research Institute, Seoul National University College of Medicine, Seoul, 03080 Korea

**Keywords:** ADAM12, ADAM17, ADAM10, Gastric cancer, Biomarker, Gastroenterology, Oncology

## Abstract

No biomarker can effectively screen for early gastric cancer (EGC). Players in the A disintegrin and metalloproteinase (ADAM)-natural killer group 2 member D (NKG2D) receptor axis may have a role for that. As a proof-of-concept pilot study, the expression of ADAM8, ADAM9, ADAM10, ADAM12, ADAM17, and major histocompatibility complex (MHC) class I chain-related sequence A (MICA), a ligand for NKG2D, in gastric cancer was investigated in silico using The Cancer Genome Atlas (TCGA) database. Subsequently, the mRNA and protein expression levels of these markers except ADAM8 were tested in blood samples from patients with EGC and healthy controls. In the TCGA data analyses, EGC tissues (*n* = 57) expressed significantly higher mRNA levels of *ADAM8*,* ADAM9*,* ADAM10*,* ADAM12*, and *ADAM17* than normal tissues (*n* = 35) (*p* < 0.005). In human blood sample analyses, *ADAM12 (p =* 0.0007), ADAM*17* mRNA (*p* < 0.0001) and ADAM10 (*p* < 0.001^7^) protein were significantly elevated in patients with EGC (*n* = 27 for mRNA and *n* = 25 for protein) compared to the controls (*n* = 30 for mRNA and *n* = 26 for protein). Areas under the curves calculated by receiver-operating characteristic analysis for *ADAM12*,* ADAM17* mRNA and ADAM10 protein were 0.7568 (95% confidence interval [CI]: 0.6334 to 0.8802), 0.8062 (95% CI: 0.6889 to 0.9234; *p* < 0.0001), and 0.8108 (95% CI: 0.6895 to 0.9320; *p* = 0.0001), respectively. Thus, *ADAM12*,* ADAM17* mRNA and ADAM10 protein levels in peripheral blood could hold potential as biomarkers for screening EGC, and further investigations are required.

**Supplementary Information:**

The online version contains supplementary material available at 10.1038/s41598-024-84237-y.

## Introduction

Gastric cancer is the fifth most common cancer and the fourth most common cause of cancer death^1^. The survival of patients with gastric cancer differs widely depending on the stage. The 5-year survival rate of patients with gastric cancer is over 70% for localized tumors, 30% for regional tumors, and less than 5% for tumors with distant metastasis^2^. Furthermore, recurrence rate is 4.0% after endoscopic resection and 0.8% after surgical resection of early gastric cancer (EGC)^3^. Thus, early detection is critical for survival, and continuous effort to detect recurrence early even after resection is required. There is no noninvasive biomarker that can effectively screen for early-stage gastric cancer. Current screening methods for gastric cancer rely on endoscopic examinations. Although endoscopic examination is very safe, potential risks due to its invasiveness necessitate the development of noninvasive biomarkers.

A disintegrin and metalloproteinase (ADAM)-natural killer group 2 member D receptor (NKG2D) axis may serve as a new source for cancer diagnostics. NKG2D, an activating receptor of natural killer (NK) cells, and its ligands, NKG2DL, expressed on cancer cells function as an immune checkpoint where cancer cells should be inspected^4^. If the NKG2D recognizes NKG2DL, NK cells send out a killing signal, which leads to apoptosis of cancer cells^4^. To evade this, cancer cells upregulate the expression of ADAMs^4^. ADAMs cleave NKG2DL into a soluble form^4^. This soluble NKG2DL, in turn, binds with NKG2D causing downregulation of NKG2D^4^. In this way, cancers evade NK cell’s immune surveillance^4^.

Previous studies have demonstrated that ADAM expression is upregulated in gastric cancer. In gastric cancer tissues, the protein expression of ADAM9, ADAM10, ADAM12, and ADAM17 was increased^5^. In metastatic lymph nodes of gastric cancer, the protein expression of ADAM8, ADAM9, ADAM10, and ADAM17 was increased^5^. In addition, the role of ADAMs is vital for gastric cancer. As an example, *ADAM9* mRNA expression and ADAM9 protease activity were increased in gastric cancer cell lines^6^. When gastric cancer cells were knocked down with si-*ADAM9* RNA or inhibited by ADAM9 blocking antibody, cancer cell progression was significantly suppressed^6^. In an animal model of gastric cancer, miR-129-5p suppressed ADAM9 protein expression and tumor growth^7^.

In a few clinical studies, the potential roles of ADAMs and MHC class I polypeptide-related sequence A(MICA), an NKG2DL, as cancer-screening biomarkers have been demonstrated. ADAM8 protein in peripheral blood was significantly increased in patients with EGC compared to healthy normal controls^8^. Moreover, the levels were observed to increase gradually from healthy controls to gastric dysplasia, EGC, and AGC^8^. It revealed a statistically significant difference between patients with EGC and normal controls. Another study showed that *ADAM9* mRNA expression was elevated in the peripheral blood of patients with early-stage hepatocellular carcinoma compared to healthy controls^9^. A soluble form of MICA that was resulted by ADAM-mediated shedding was associated with prognosis of patients with gastric cancer; patients with gastric cancer having a higher level of soluble MICA had a lower survival rate^10^. Moreover, soluble MICA was significantly increased in patients with gastric cancer compared with healthy controls^10^.

Based on these previous studies, we postulated that ADAMs and MICA have potential as biomarkers for screening for gastric cancer. In this pilot study, we aimed to test this hypothesis. First, we characterized ADAM expression in gastric cancer by using bioinformatic techniques. Second, we validated the results by analyzing human blood samples. We hope the results of this study will provide a direction where the future investigations for biomarkers for early-stage cancers should be headed to.

## Methods

### Study design

This pilot study evaluated the association between ADAMs, MICA, and gastric cancer. In a previous study, ADAM9, ADAM10, and ADAM17 expression was increased in both primary cancer tissues and metastatic lymph node tissues of gastric cancer^5^. In another study, a soluble form of MICA was associated with the prognosis of patients with gastric cancer^10^. Thus, ADAM9, ADAM10, ADAM17, and MICA were chosen as study markers.

For the initial assessment of target markers, we performed an in-silico analysis using The Cancer Genome Atlas (TCGA) database. Subsequently, quantitative real-time polymerase chain reaction (qRT-PCR) and enzyme-linked immunosorbent assay (ELISA) were performed on human blood samples.

After the initially planned study, we expanded the bioinformatic analyses to cover all ADAMs and NKG2DLs. In this process, we found that ADAM12 had statistical significance. Thus, we added ADAM12 as a target marker in the blood sample analysis. Also, we identified that ADAM8 had statistical significance. Thus, we included ADAM8 in the in-silico analysis, but did not include it in the blood sample analysis for there is already a study that investigated the association between serum ADAM8 protein and gastric cancer^8^.

### In silico analysis with TCGA database

We downloaded the transcriptomic, survival, and clinical data of 415 patients with GC (indexed as STAD) from the Xena TCGA database hub (https://xenabrowser.net) generated by the University of North Carolina TCGA Genome Characterization Center. Transcriptomic data were acquired from 415 gastric cancer tissues indexed as primary tumors and 35 nearby normal tissues indexed as solid normal tissues.

### Participants

This study was conducted at Seoul National University Hospital and Chaum Health Check-up Center between July 2021 and March 2023. Patients who recently received a diagnosis of gastric cancer at Seoul National University Hospital were included in the gastric cancer group. Gastric cancer was confirmed using endoscopic and histopathological examinations. Clinical stage was determined using endoscopic ultrasonography and computed tomography. For the control group, healthy individuals who were found to have no evidence of cancer during a health check-up at the Chaum Health Check-up Center were included. Written informed consent for enrollment was obtained. This study was approved by the Institutional Review Board (IRB) of Seoul National University Hospital (IRB number: 2102-189-1203) and CHA Bundang Medical Center (IRB number: 2021-07-073). This study was conducted in accordance with the Declaration of Helsinki.

Upon enrollment, 10 mL of peripheral blood was drawn to one SST bottle (BD Vacutainer^®^, 5 mL) and one EDTA bottle (BD Vacutainer^®^, 5 mL). The serum and plasma samples were stored at − 80 °C. The serum samples were used for protein quantification with ELISA, and the plasma samples were used for mRNA quantification for qRT-PCR.

### Population size calculation

To calculate the required population size, we used a previous study that evaluated the association between ADAM8 protein and gastric cancer as a reference study^8^. As a primary endpoint, we aimed to find a marker that can distinguish EGC patients from healthy controls. Thus, we used the average value of serum ADAM8 protein in normal controls (1.7 ng/mL) and patients with EGC (53.9 ± 36.9 ng/mL). Using an alpha of 0.05, power of 80%, and enrollment ratio of 1, the population size (healthy controls: patients with EGC) of the training set was calculated to be 16 (8:8). Assuming that sample handling errors may occur in 20% of cases, the population size was determined to be 20 (10:10). If the training study yielded significant results, a validation study was planned to follow with a population size of 40 (20:20) was conducted. As a secondary endpoint, we aimed to determine the differences between patients with advanced gastric cancer (AGC) and healthy controls. The population size for AGC was planned to be the same as that for EGC. The secondary endpoint study was terminated when the primary endpoint was met.

### mRNA isolation and real-time PCR

Total RNA was extracted from the cells using the RNeasy Mini Kit (Qiagen, Hilden, Germany) following the manufacturer’s instructions. The purity and concentration of the isolated RNA was assessed using a Nanodrop-1000 spectrophotometer (Thermo Fisher Scientific). Subsequently, 1 µg of RNA was reverse transcribed into cDNA using the AccuPower CycleScript RT Premix (BIONEER, Daejeon, Korea) according to the manufacturer’s protocol. qRT-PCR was performed on a CFX96 Touch real-time PCR detection system (Bio-Rad Laboratories, Hercules, CA, USA) using iQ™ SYBR Green Supermix (BIONEER). The specific primer sets used are listed in **Supplementary Table ****S1**. The following PCR reactions were performed: initial denaturation (95 °C for 10 min) followed by 45 cycles of denaturation (95 °C for 10 s) and annealing (55 °C for 30 s). The raw data obtained from qRT-PCR were analyzed using the 2^-ΔΔCt^ method, and the expression levels were normalized to the reference gene GAPDH as an endogenous internal control. All experiments were performed in duplicates.

### Enzyme-linked immunosorbent assay

ELISA kits were used for the detection of ADAM9 (Aviva Systems Biology, San Diego, CA, USA), ADAM10 (Novus Biologicals, Centennial, CO, USA), ADAM12 (R&D Systems Inc., Minneapolis, MN, USA), ADAM17 (Novus Biologicals), and MICA (Invitrogen, Waltham, MA, USA). ELISA was performed according to the manufacturer’s protocol. The absorbance was measured at an appropriate wavelength using a microplate reader (Biotek, Winooski, Vermont, USA). Standard curves were generated using known concentrations of recombinant proteins to quantify the target protein levels in serum samples. All experiments were performed in duplicates.

### Statistical analysis

The statistical significance of differences between the groups was determined using Shapiro–Wilk test, Student’s *t*-test, Mann–Whitney U test, paired *t*-test, and analysis of variance (ANOVA). A *p*-value of < 0.05 was considered statistically significant. Statistical analyses of TCGA dataset were performed using R software (https://www.r-project.org/, Version 4.3.1). The “ggplot2” package (Version 3.4.3) was used for data summary and analysis. For the evaluation of diagnostic performance, receiver-operating characteristic (ROC) curve analysis was performed to calculate the area under the curve (AUC). Graphs were generated using Graph pad Prism9 (Graph Software, La Jolla, California, USA).

## Results

### Part 1. *ADAM* and *MICA* mRNA expression in gastric cancer tissues from TCGA database

To evaluate the expression levels of *ADAM* and *MICA* mRNAs in gastric cancer tissues, we performed in-silico analyses of 415 gastric cancer tissues and 35 normal tissues from TCGA database.

#### *ADAM* and *MICA* mRNA expression in gastric cancer

**Fig. 1:**
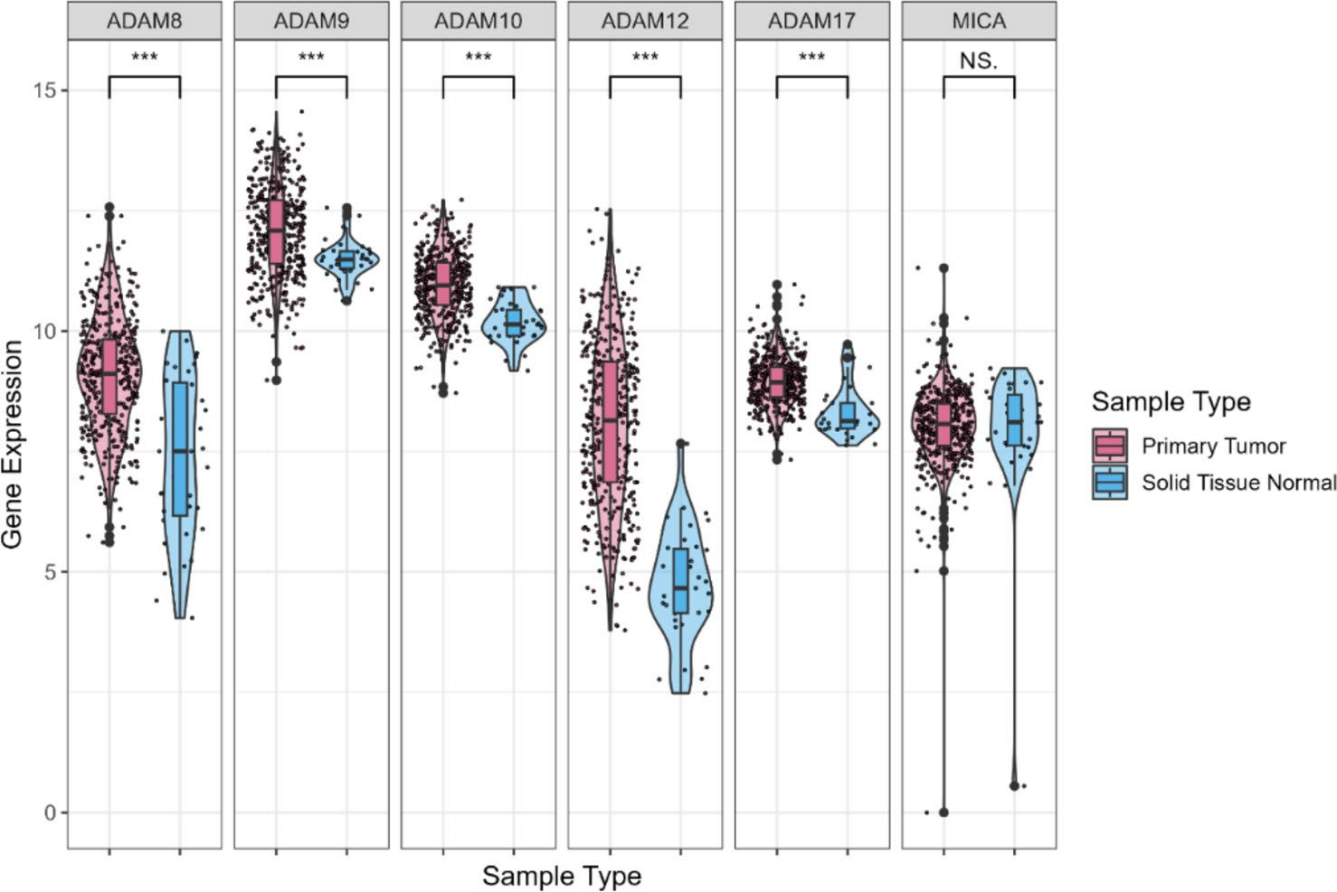
*ADAM* and *MICA* mRNA expression in gastric cancer tissues compared with normal tissues from TCGA database. Expression levels of *ADAM* and *MICA* mRNAs were compared in gastric cancer tissues (*n* = 415) and normal tissues (*n* = 35) via in silico analyses using TCGA database. *p < 0.05, **p < 0.005, ***p < 0.001, NS. not specific; t-test.

Gastric cancer tissues (*n* = 415) expressed significantly higher mRNA levels of *ADAM8*,* ADAM9*,* ADAM10*,* ADAM12*, and *ADAM17* (*p* < 0.001) than normal tissues (*n* = 35) (Fig. 1). When gastric cancer tissue was paired with normal tissue from the same patient with gastric cancer (*n* = 32), *ADAM8*,* ADAM9*,* ADAM10*,* ADAM12*, and *ADAM17* mRNAs showed significantly higher expression in gastric cancer tissues (*p* < 0.05) (**Supplementary Figure ****S1**).

#### *ADAM* and *MICA* mRNA expression in early gastric cancer

**Fig. 2:**
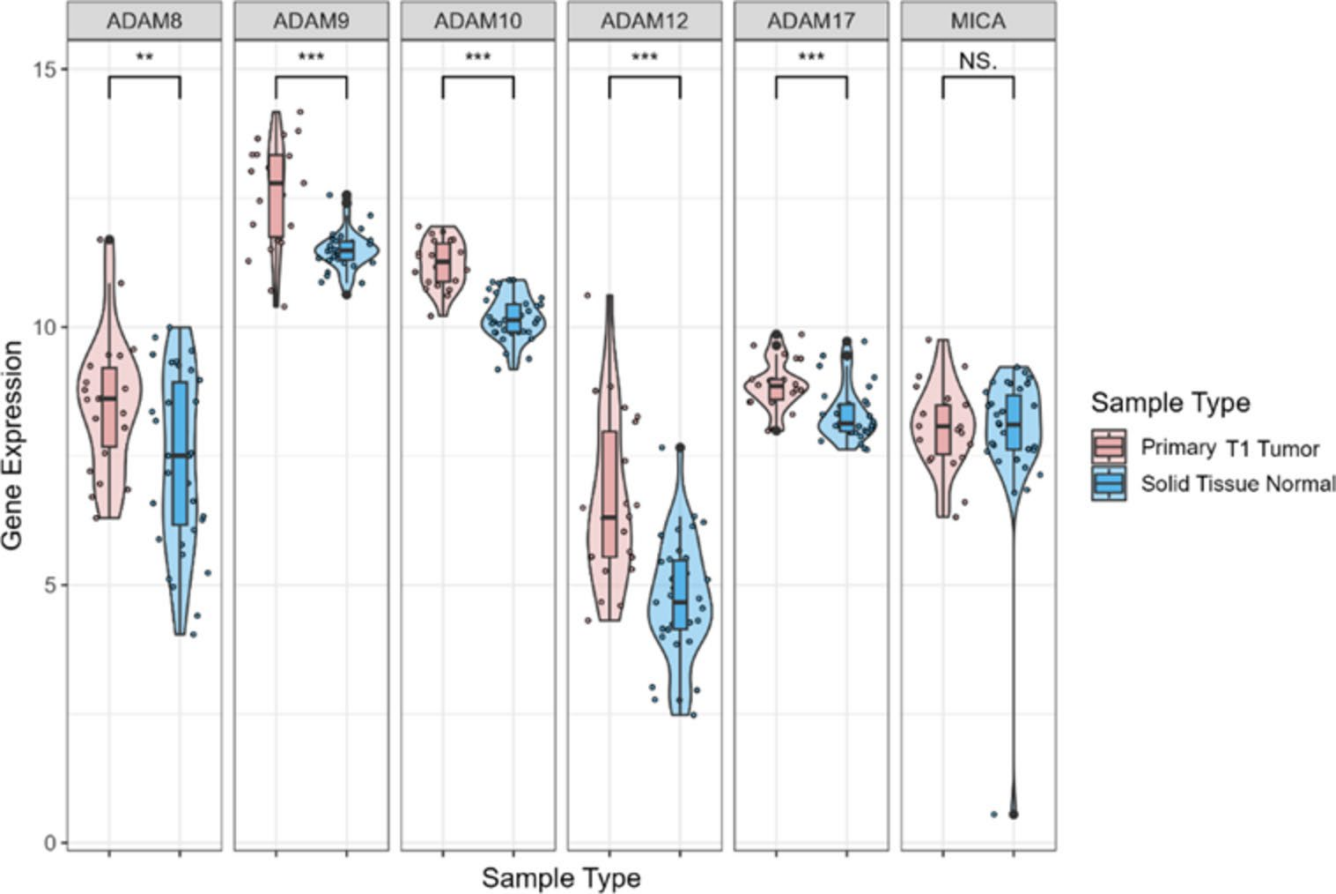
*ADAM* and *MICA* mRNA expression in early gastric cancer tissues compared with normal tissues from TCGA database. mRNA expression levels of *ADAM* and *MICA* were compared between gastric cancer tissues with T1 stage (*n* = 22) and normal tissues (*n* = 35) via in silico analyses using TCGA database. *p < 0.05, **p < 0.005, ***p < 0.001, NS. not specific; t-test.

To determine whether the above association was observed in EGC, gastric cancer tissues from T1 stage tumors were grouped as EGC (*n* = 22) and compared with normal tissues (*n* = 35). EGC expressed significantly higher mRNA levels of *ADAM8*,* ADAM9*,* ADAM10*,* ADAM12*, and *ADAM17* than normal tissues (*p* < 0.005) (Fig. 2).

#### *ADAM* and *MICA* mRNA expression according to gastric cancer stage

**Fig. 3:**
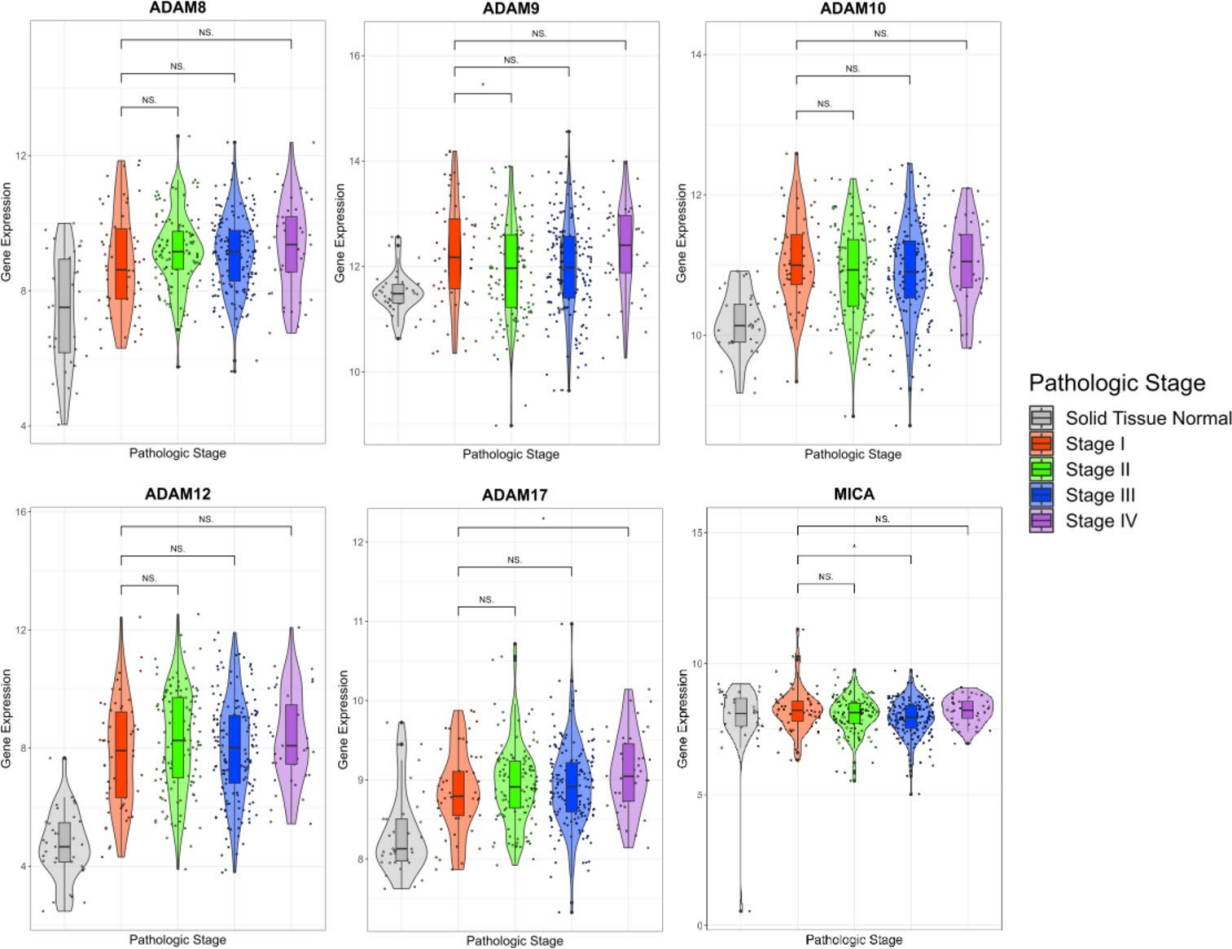
*ADAM* and *MICA* mRNA expression in normal and gastric cancer tissues according to gastric cancer stage in TCGA database. *ADAM* and *MICA* mRNA expression in normal (*n* = 35) and gastric cancer tissues according to gastric cancer stage groups (stage I, *n* = 57; stage II, *n* = 123; stage III, *n* = 171; stage IV, *n* = 41) is depicted. The difference of *ADAM* and *MICA* mRNA expression was compared between stage I gastric cancer group and other stage groups. *p < 0.05, **p < 0.005, ***p < 0.001, NS. not specific; t-test.

*ADAM* and *MICA* mRNA expression in normal (*n* = 35) and gastric cancer tissues according to gastric cancer stage (stage I, *n* = 57; stage II, *n* = 123; stage III, *n* = 171; stage IV, *n* = 41) is shown in Fig. 3. *ADAM* and *MICA* mRNA expression in each group of stage II, III and IV gastric cancer was compared with that in stage I gastric cancer group, one by one. A statistically significant difference (*p* < 0.05) without clinical significance was observed in a few parts of the analysis: *ADAM17* between stages I and IV, *ADAM9* between stages I and II, *MICA* between stages I and III.

### Part 2. ADAM and MICA expression in the peripheral blood of patients with gastric cancer

#### Population characteristics

The characteristics of the study population are described in Table 1. Eleven patients with EGC, 3 with AGC, and 12 healthy controls were enrolled in the training set. Among these, two EGC, one AGC, and two control samples were inadequate owing to hemolysis, coagulation, and insufficient amounts. Thus, nine patients with EGC, two patients with AGC, and ten healthy controls were included in the analysis. In the validation set, 20 patients with EGC, 5 patients with AGC, and 22 healthy controls were enrolled. Among them, two EGC samples and two control samples were inadequate. Thus, 18 patients with EGC, 5 patients with AGC, and 20 healthy controls were included in the analysis. Before we completed enrollment of patients with AGC, the primary endpoint was met; thus, further enrollment of patients with AGC was terminated. Sample handling errors occurred in two AGC serum samples in the training set and in two EGC and three control serum samples in the validation set.

**Table 1:** Population characteristics.

Training set		Validation set	
Early gastric cancer (*n* = 9)		Early gastric cancer (*n* = 18)	
Men	8 (88.9)	Men	16 (88.9)
Age	72.8 ± 7.14	Age	60.9 ± 11.6
Histology		Histology	
WD	5 (55.6)	WD	6 (33.3)
MD	3 (33.3)	MD	6 (33.3)
PD	0	PD	1 (5.56)
SR	1 (11.1)	SR	5 (27.8)
Stage		Stage	
cT1aN0	5 (55.6)	cT1aN0	7 (38.9)
cT1bN0	3 (33.3)	cT1bN0	11 (61.1)
Advanced gastric cancer (*n* = 2)		Advanced gastric cancer (*n* = 5)	
Men	2 (100)	Men	3 (60)
Age	74.5 ± 0.707	Age	58.8 ± 5.63
Histology		Histology	
WD	0	WD	0
MD	1 (50)	MD	2 (40)
PD	1 (50)	PD	1 (20)
SR	0	SR	2 (40)
Stage		Stage	
cT2N0	2 (100)	cT2N0	2 (40)
		cT3N0	3 (60)
Control (*n* = 10)		Control (*n* = 20)	
Men	5 (50)	Men	9 (45)
Age	47.5 ± 8.53	Age	44.2 ± 10.8

Values are presented in number (%) or mean ± standard deviation.

*WD* well-differentiated tubular adenocarcinoma, *MD* moderately differentiated tubular adenocarcinoma, *PD* poorly differentiated tubular adenocarcinoma, *SR* poorly cohesive carcinoma with signet ring cells.

#### Training set

In the mRNA quantification with the peripheral blood sample, patients with EGC expressed significantly higher mRNA levels of *ADAM10* (*p* = 0.031), *ADAM12* (*p* < 0.0001), *ADAM17* (*p =* 0.0279) and *MICA* (*p* = 0.0334) than the healthy controls (**Supplementary Figure S2**). *ADAM9* (*p* = 0.0789) mRNA level showed an increasing trend.

In protein quantification, ADAM17 (*p* = 0.084) protein showed an increasing trend in EGC compared to that in the controls (**Supplementary Figure S3**). ADAM9 protein was not detected. Therefore, further investigation of ADAM9 protein was terminated. ADAM12 protein had an outlier (3201.1 pg/mL) in the EGC group that was too high than that in the other EGC samples (0–490.1 pg/mL). The inclusion or exclusion of this sample did not affect statistical significance (*p* = 0.0204 and *p* = 0.039, respectively). **Supplementary Figure S3** excludes this outlier value.

#### Validation set

In the mRNA quantification, patients with EGC expressed significantly higher mRNA levels of *ADAM12* (*p* = 0.0023) and *ADAM17* (*p <* 0.0001) than the controls (**Supplementary Figure S4**).

In protein quantification, ADAM10 (*p* < 0.001) protein was significantly increased in patients with EGC compared to that in the controls (**Supplementary Figure S5**). ADAM12 protein had an outlier (122201.9 pg/mL) in the control group that was too high than that in the other control samples (0-209.2 pg/mL). Including (*p* = 0.393) and excluding (*p* = 0.005) this sample had a statistically significant effect. **Supplementary Figure S5** excludes this outlier value.

#### Combined analyses

When the training and validation sets were combined, there were 27 patients with EGC, 7 with AGC, and 30 controls. Analyses were performed on the combined population.

**Fig. 4:**
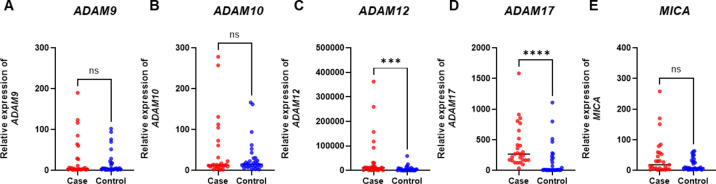
*ADAM* and *MICA* mRNA expression in the combined sets of plasma samples of patients with early gastric cancer and healthy controls. mRNA expression levels of (**A**) *ADAM9* (*p* = 0.858), (**B**) *ADAM10* (*p* = 0.803), (**C**) *ADAM12* (*p* = 0.0007), (**D**) *ADAM17* (*p* < 0.0001), and (**E**) *MICA* (*p* = 0.319) were examined using qPCR in plasma samples obtained from early gastric cancer (case, *n* = 27, shown in red) and healthy controls (control, *n* = 30, shown in blue). The data were normalized to an internal control (GAPDH) and presented as relative expression. *p < 0.05, **p < 0.005, ***p < 0.001, ****p < 0.0001, ns. not specific; t-test or Mann–Whitney U test.

First, the EGC group was compared with the control group. In mRNA quantification, patients with EGC (*n* = 27) expressed significantly higher mRNA levels of *ADAM12* (*p* = 0.0007) and *ADAM17* (*p* < 0.0001) than the controls (*n* = 30) (Fig. 4).

**Fig. 5:**
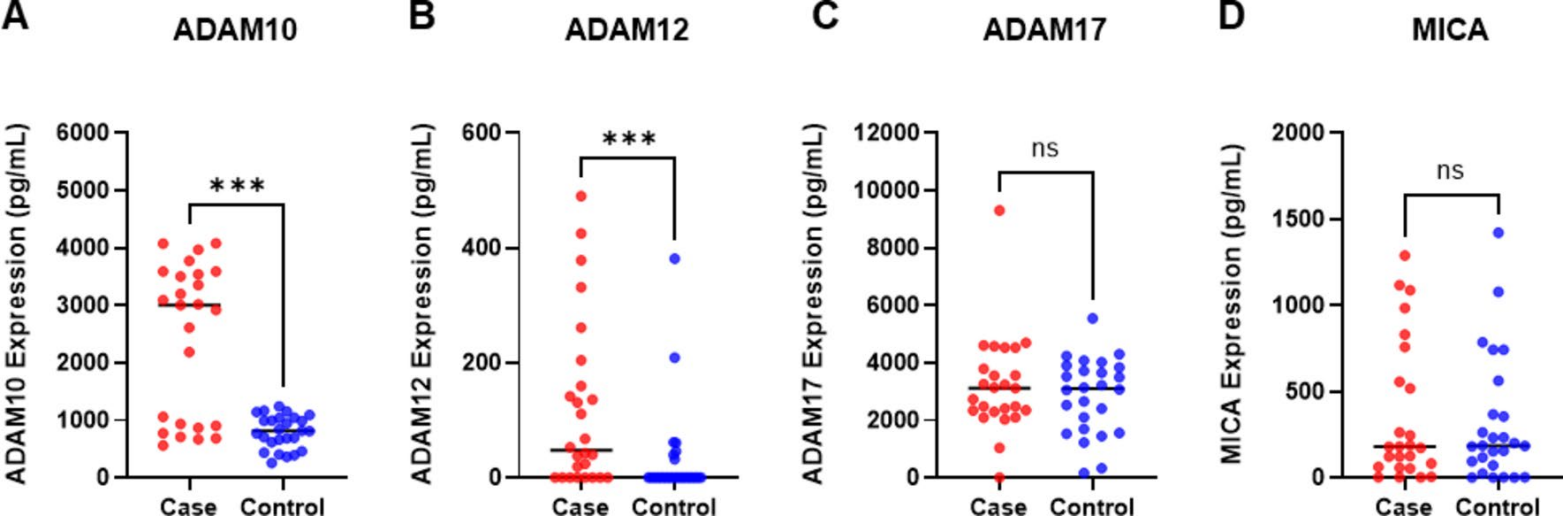
ADAM and MICA protein expression in the combined sets of serum samples of patients with early gastric cancer and healthy controls. Expression levels of (**A**) ADAM10 (*p* < 0.001), (**B**) ADAM12 (*p* < 0.001), (**C**) ADAM17 (*p* = 0.702), and (**D**) MICA (*p* = 0.812) were examined using ELISA in serum samples obtained from patients with early gastric cancer (case, *n* = 25 [*n* = 24 for ADAM12], shown in red) and healthy controls (control, *n* = 26 [*n* = 25 for ADAM12], shown in blue). *p < 0.05, **p < 0.005, ***p < 0.001, ****p < 0.0001, ns. not specific; t-test or Mann–Whitney U test.

In protein quantification, ADAM10 (*p* < 0.001) protein was significantly increased in EGC samples (*n* = 25) compared to that in the controls (*n* = 26) (Fig. 5). For ADAM12 protein analysis, two outliers were excluded: one EGC sample from the training set and one control sample from the validation set. ADAM12 (*p* < 0.001) protein levels were significantly higher in patients with EGC (*n* = 24) than in healthy controls (*n* = 25). Figure 5 was drawn after excluding two outliers.

Second, the AGC group (*n* = 7) was compared with the control group (*n* = 30). In the mRNA quantification, patients with AGC (*n* = 7) expressed significantly higher mRNA levels of *ADAM17* (*p* = 0.003) than the controls (*n* = 30) (**Supplementary Figure S6**).

The protein levels of ADAM10 (*p* = 0.006) and ADAM12 (*p =* 0.014) were significantly increased in patients with AGC (*n* = 5) than in controls (*n* = 26) (**Supplementary Figure S7**). For ADAM12 protein analysis, one outlier in the control sample was excluded (*p* = 0.751). The protein level of MICA (*p* = 0.042) was significantly decreased in patients with AGC than in controls (**Supplementary Figure S7**).

#### Diagnostic performance of *ADAM12*,* ADAM17* mRNA and ADAM10 protein

**Fig. 6:**
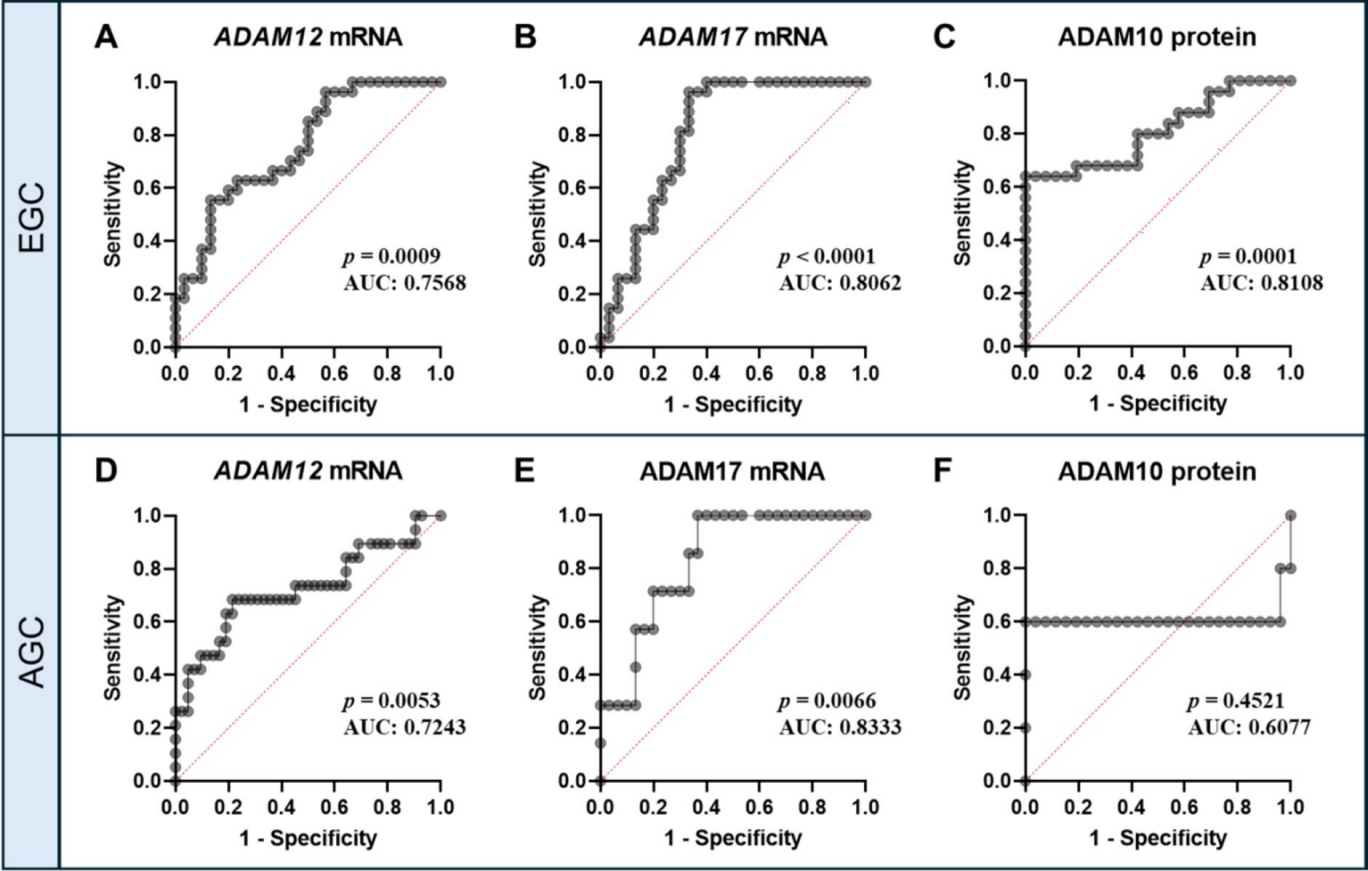
Receiver operating characteristic (ROC) curves and areas under the curves (AUC) of *ADAM12*,* ADAM17* mRNA and ADAM10 protein for the prediction of early gastric cancer (EGC) and advanced gastric cancer (AGC) in the combined population. Panels (**A**)–(**C**) represent the analyses in the EGC, and (**D**)–(**F**) in the AGC. The combined population of training set and validation set for analyses of EGC comprised of EGC (*n* = 27) and controls (*n* = 30) for the mRNAs and EGC (*n* = 25) and controls (*n* = 26) for the protein. The combined population for analyses of AGC comprised of AGC (*n* = 7) and controls (*n* = 30) for the mRNAs and AGC (*n* = 5) and controls (*n* = 26) for the protein.

Diagnostic performance of *ADAM12*,* ADAM17* mRNA in EGC (*n* = 27) and controls (*n* = 30) and ADAM10 protein in EGC (*n* = 25) and controls (*n* = 26) of the combined population was evaluated with ROC analysis. *ADAM12* mRNA had an AUC of 0.7568 (95% confidence interval [CI]: 0.6334 to 0.8802; *p* = 0.0009), *ADAM17* mRNA had an AUC of 0.8062 (95% CI: 0.6889 to 0.9234; *p* < 0.0001), and ADAM10 protein had an AUC of 0.8108 (95% CI: 0.6895 to 0.9320; *p* = 0.0001) (Fig. 6A, B, C). For the purpose of comparison, the diagnostic performance of *ADAM12* and *ADAM17* mRNA in AGC (*n* = 7) and controls (*n* = 30) and ADAM10 protein in AGC (*n* = 5) and controls (*n* = 26) was additionally presented in Fig. 6. *ADAM12* mRNA had an AUC of 0.7243 (95%CI: 0.5716 to 0.8770; *p* = 0.0053), *ADAM17* mRNA had an AUC of 0.8333 (95% CI: 0.6949 to 0.9718; *p* = 0.0066), and ADAM10 protein had an AUC of 0.6077 (95% CI: 0.1859 to 1.000; *p* = 0.4521) (Fig. 6D, E, F). Although the diagnostic performance decreased in AGC due to the small sample size, the trend is consistent with that in EGC.

## Discussion

This proof-of-concept study tested whether players in the ADAM-NKG2D axis have clinical applicability in gastric cancer diagnostics. In this study, we found that the mRNA expression levels of *ADAM8*,* ADAM9*,* ADAM10*,* ADAM12*, and *ADAM17* were significantly higher in EGC tissues than in normal tissues. Among these, we found that *ADAM12*, *ADAM17* mRNA and ADAM10 protein levels were significantly increased in the peripheral blood of patients with EGC compared to those in controls revealing fair performance. This result implies that *ADAM12*, *ADAM17* mRNA and ADAM10 protein have potential as biomarkers for screening EGC.

The incidence of cancer is increasing with an aging population. The most effective cancer management strategies are early detection and complete excision. Health-checkups are useful for early detection but have shortcomings in terms of risks and expenses. There are multiple tumor markers, but only a few have proven useful in cancer screening^11^. Thus, most tumor markers are used to assess treatment response rather than to screen for cancer. Hence, the development of a safe, noninvasive method for checkups, such as blood tests for cancer screening, has been pursued^11^. A major hurdle in developing a cancer-screening biomarker is finding a marker with sufficient power to detect cancer at an early stage.

To solve the problem of detecting early-stage cancers, liquid biopsy is under investigation. Liquid biopsy examines cancer-derived materials such as circulating tumor cells, exosomes, extracellular vesicles, and cell-free nucleic acids^12^. In addition, algorithmic analysis of cancer risk with a composite of various markers are being investigated. For example, CancerSEEK calculated the risks of eight cancers using 16 circulating tumor DNAs and eight proteins^13^. Circulating tumor DNAs are not easily detected in early-stage cancers^14,15^. To address this problem of detecting early-stage cancers, the authors commented that protein markers were added^13^. The CancerSEEK could detect cancers of the ovary, liver, stomach, pancreas, esophagus, colorectum, lung, and breast with a sensitivity of 70%. Although CancerSEEK has achieved significant advancements in cancer diagnostics, the detection of early-stage cancers remains a limitation. This limitation highlights the importance of ongoing research in the development of novel biomarkers.

Therefore, the use of ADAMs warrants further investigation. The ADAM family belongs to the superfamily of zinc-dependent metalloproteinases, and are expressed in various tissues in body^16^. Currently, 22 ADAM members were discovered in humans^17^. Among those, 12 ADAMs (ADAM8, 9, 10, 12, 15, 17, 19, 20, 21, 28, 30, and 33) are proteolytically active containing the catalytic metalloproteinase domain^17–19^. ADAMs are often called as a sheddase involved in ectodomain shedding. They proteolytically cleave membrane-bound proteins such as cytokines, growth factors, receptors, ligands and cell adhesion molecules, which result in important biological processes such as cell signaling, cell adhesion, migration, proteolysis, and tissue remodeling^17,20^. It is known that ADAM-mediated shedding can be constitutive or induced by signaling through G-protein coupled receptor (GPCR) activators, protein kinase C (PKC) activators, calcium ionophores, and others^20^. The normal function of ADAMs is best demonstrated in knockout studies. ADAM knockout in mice resulted in infertility, developmental failure of heart and nervous system, muscle defect, and so on^20^. For instance, apical surface of uterine epithelium is covered by mucins. A transmembrane mucin MUC1 exerts anti-adhesive effect thus serving as a physical barrier against microbial attack, but this barrier needs to be overcome when the time of blastocyst attachment comes. There is evidence that ADAMs, especially ADAM17, take part in the process of clearing of MUC1 by shedding it locally on the uterine epithelium to facilitate embryo implantation^17,21,22^.

On the other hand, dysregulation of ADAMs are involved in several pathologic processes such as cancer, cardiovascular disease, asthma and Alzheimer’s disease^20^. Especially, ADAMs are closely linked to cancer development and metastasis^16,19^. Overexpression of ADAMs was observed in many cancers^23–28^, and higher expression was associated with poor prognosis^23,25,29–31^. Knocking-down ADAMs reduced cancer cell proliferation and invasion in vitro and tumor growth in vivo^32–34^. Furthermore, knocking-down ADAMs increased chemosensitivity and radiosensitivity of cancer cells^35–37^. Inhibiting ADAMs with specific molecules such as monoclonal antibody reduced tumor growth, increased chemosensitivity, and decreased shedding of NKG2DL leading to enhanced immune recognition by NK cells^38–40^. These observations support the idea that ADAMs may serve as diagnostic and therapeutic targets for cancer.

In the current study, we identified ADAM10, *ADAM12* and *ADAM17* simultaneously as potential biomarkers for gastric cancer diagnosis. This finding is consistent with the findings of other studies showing that the expression of ADAM10, ADAM12 and ADAM17 is increased in gastric cancer tissues, and that increased expression of those in gastric cancer tissues is associated with poor survival in patients with gastric cancer^5,41–46^. ADAM10, ADAM12 and ADAM17 are known to have very similar sequences and crystal structures^17,47,48^. Consequently, they have similar functions sharing some substrates such as pro-heparin binding (HB)-epidermal growth factor (EGF)^17,47^.

ADAM10 and ADAM17 were often studied together having the greatest number of substrates and shared substrates among ADAM members^17^. The expression of ADAM10 and ADAM17 was increased in biopsy specimens of non-cancerous gastric mucosa infected with *Helicobacter pylori*, the well-known carcinogen for gastric cancer, compared to those in specimens not infected^42^. When gastric epithelial cells were infected with *H. pylori*, *ADAM10* and *ADAM17* mRNA expression demonstrated a temporal increase^42^. In addition, ADAM10 and ADAM17 expression upon *H. pylori* infection may explain the paradox that higher expression of MUC1, a protective barrier on the gastric mucosa against *H. pylori* attachment^49^, is actually associated with poor prognosis of gastric cancer^50^. That is, ADAM17 mediated MUC1 shedding induced by *H. pylori* infection may have caused increased expression of MUC1 as a coping mechanism although it failed to prevent gastric cancer development. Another study demonstrated that *H. pylori* infection of gastric epithelial cells caused phosphorylation of ADAM17 C-terminal potentiating the cleavage function of ADAM17 to the extent that shedding of HB-EGF could lead to EGF receptor transactivation^51^. These findings indicate that gastric carcinogenesis caused by chronic infection with *H. pylori* is mediated by aberrant expression of ADAM10 and ADAM17.

There are other ways in which ADAM10 and ADAM17 participate in gastric cancer development via various signaling pathways. Neurogenic locus notch homolog (Notch) and Wingless-related integration site (Wnt) signaling pathways had positive correlation with ADAM17 expression in gastric cancer^5^. Erythropoietin-producing hepatocellular (Eph) receptor type A8 (EphA8) expression was associated with poor prognosis of patients with gastric cancer, and its knockdown decreased the expression of ADAM10, indicating that EphA8 is an upstream regulator of ADAM10^52^. In gastric cancer cells, IL-8 induced shedding of EGFR ligands to lead to EGFR transactivation in a pathway that is dependent on ADAM10 but not on ADAM12 or ADAM17^53^. Chemokine (C-X-C motif) ligand 16 (CXCL16) and C-X-C motif chemokine receptor 6 (CXCR6) axis that activate protein kinase B (Akt) and MAPK signaling pathways in gastric carcinogenesis was dependent on ADAM10^54^. Micro RNAs are also involved in gastric cancer development by influencing ADAM10 or ADAM17. The expression levels of miR-448 and miR-320a were negatively correlated with those of ADAM10 in gastric cancer tissues, and overexpression of these miRNAs suppressed gastric cancer cell proliferation, colony formation, and invasion^55,56^. Overexpression of miR-338-3p, which is regulated by the circular RNA circ_0051620, inhibited gastric cancer cell migration and invasion by inhibiting ADAM17^57,58^.

Compared to ADAM10 and ADAM17, the role of ADAM12 in relation to gastric cancer development is relatively less studied. There is evidence that ADAM12 is involved in epithelial-mesenchymal transition and metastasis^44^. A study suggested that ADAM12 facilitate tumor progression by promoting metastasis and immune infiltration in gastric cancer by showing that genes involved with extracellular matrix and tumor microenvironment were associated with ADAM12 in gene enrichment analyses^59^. Also, a proteomic study revealed that ADAM12S, a secreted form of ADAM12, promotes migration of gastric cancer cells by upregulating CD146, a cell adhesion molecule dependent on the catalytic residue of ADAM12S^60^. In terms of micro RNA, one study showed that miR-30c-5p was downregulated while its target, ADAM12, was upregulated in gastric cancer tissues^61^. Although the mechanistic role of ADAM12 in gastric carcinogenesis has not been sufficiently elucidated yet, there were already two studies demonstrating that ADAM12 was increased in the urine samples of gastric cancer patients compared to controls suggesting that urinary ADAM12 holds potential as a biomarker for gastric cancer^62,63^. On the other hand, ADAM12 may play a crucial role in relation to human epidermal growth factor receptor 2 (HER2) in gastric cancer. Overexpression of HER2 is involved in the pathogenesis of gastric cancer, and HER2 positivity ranges from 6.0 to 29.5% in gastric cancer^64^. A study in head and neck cancer cells demonstrated that ADAM12 increased HER2 expression, HER2 inhibition decreased ADAM12 expression, and HER2 transfection increased ADAM12 expression suggesting there is a positive feedback loop between those two^65^.

Above studies support that *ADAM12*, *ADAM17* and ADAM10 hold potential as biomarkers for gastric cancer. We specifically demonstrated their potential in screening EGC. In general, it is easier to demonstrate significant differences between patients with advanced cancer and healthy controls. However, demonstrating the differences between early-stage cancer and healthy controls is a challenging task. Thus, demonstrating significant difference of the above study markers between EGC and healthy controls is a great achievement of this study.

This study had some limitations. First of all, the sample size was very small. We calculated population size based on a reference study which evaluated the association between ADAM8 protein and gastric cancer. Since its outcome was remarkable from the training set, the population size was calculated quite small as was described in the methods. We think inconsistency between the training set and validation set, which happened in ADAM10, was caused by the small sample size. Secondly, age and sex matching could not be done in this study. Planning a pilot-study like a sieve to test the potential of 10 markers altogether, we forwent the matching but enrolled participants who were willing to donate blood samples for the study. As a matter of fact, it was quite difficult to find participants because blood draw was bothersome for the participants, and younger people were more willing to donate blood samples in patients and controls alike. Thirdly, it was unfortunate that we were unable to enroll many patients with AGC. In Korea, the detection rate of AGC has significantly dropped over the last decade thanks to national cancer screening program. Most of the patients with gastric cancer are being detected at an early stage. Thus, before enrolling patients with AGC up to the initially planned number, we observed statistical significance in patients with EGC meeting the primary endpoint. Therefore, we stopped enrolling patients further. Although we could not compare healthy controls and patients with EGC and AGC in sufficiently large numbers, the analyses with TCGA data indirectly indicated that the difference between early and advanced stages may not be too large. Additionally, we did not include ADAM12 protein as a main finding of this study because ADAM12 protein had a few outliers. However, we see that ADAM12 also holds potential and is worth further study for many biomarkers in real-world also have outliers of sometimes unknown reasons. Lastly, we suggest that future studies be designed in a larger population with age and sex matching and be followed longitudinally to better characterize the study markers.

## Conclusion

In summary, we hypothesized that players in the ADAM-NKG2D axis, the less investigated targets, may have a role in gastric cancer diagnostics. Investigations first with bioinformatic analyses and second with human blood samples revealed that *ADAM12*,* ADAM17* mRNA and ADAM10 protein have a potential as biomarkers for screening EGC. This finding is particularly encouraging in that finding a biomarker with sufficient power to detect cancer at an early stage has been a substantial challenge to researchers. We believe that *ADAM12*,* ADAM17* mRNA and ADAM10 protein are worth further investigation as biomarkers for gastric cancer screening, and that players in the ADAM-NKG2D axis are worth investigation for cancer diagnostics and therapeutics.

## Electronic supplementary material

Below is the link to the electronic supplementary material.

Supplementary Material 1

## Data Availability

The data are available from the corresponding author upon reasonable request.
