# Supplementary material for: A disintegrin and metallopeptidase domain (ADAM) 12, ADAM 17 mRNA and ADAM10 protein hold potential as biomarkers for detection of early gastric cancer
Source: Sci Rep. 2025 Jan 4;15:763. doi: 10.1038/s41598-024-84237-y (PMC11700123; doi:10.1038/s41598-024-84237-y)
Supplement: Supplementary file 1 — Supplementary Material 1 [file 41598_2024_84237_MOESM1_ESM.docx]

Supplementary materials for

*A disintegrin and metallopeptidase domain (ADAM) 12, ADAM 17* mRNA and ADAM10 protein hold potential as biomarkers for detection of early gastric cancer

Short title: *ADAM12, ADAM17* mRNA and ADAM10 protein to screen EGC

Sooyeon Oh, MD, PhD^1,2, †^, Sang-Soo Lee, MSc^3, †^, Hoeyoung Jin, MSc^3, †^,
Seo-Hyeon Choi, MSc^3^, Choong-Keun Cha, MD, PhD^1^, Jooho Lee, MD, PhD^4^,
 KyuBum Kwack, PhD^3,*^, Sang Gyun Kim, MD, PhD^5,†,*^, and Sang-Woon Choi, MD, PhD^1^.

^1^ Chaum Life Center, CHA University School of Medicine, Seoul 06062, Korea.

^2^ Graduate school of Internal Medicine, Seoul National University College of Medicine, Seoul 03080, Korea.

^3^ Department of Biomedical Science, College of Life Science, CHA University, Seongnam 13488, Korea

^4^ Department of Gastroenterology and Hepatology, CHA Bundang Medical Center, CHA University School of Medicine, Seongnam 13496, Korea.

^5^ Division of Gastroenterology, Department of Internal Medicine and Liver Research Institute, Seoul National University College of Medicine, Seoul 03080, Korea.

^†^ Co-first authors with equal contribution

*Co-corresponding authors with equal contribution

Correspondence to:

1. Sang Gyun Kim, MD, PhD

Division of Gastroenterology, Department of Internal Medicine and Liver Research Institute, Seoul National University College of Medicine, Seoul 03080, Korea, Tel: +82-2-740-8112, Fax: +82-2-743-6701, E-mail: [harley@snu.ac.kr](mailto:harley@snu.ac.kr)

1. KyuBum Kwack, PhD

Department of Biomedical Science, College of Life Science, CHA University, Seongnam 13488, Korea, Tel.: +82-31-881-7141, Fax: +82-31-881-7219, E-mail: [kbkwack@cha.ac.kr](mailto:kbkwack@cha.ac.kr)

**Supplementary Table S1. Primers used for qRT-PCR**

| **Target** | **Direction** | **Sequence ( 5' → 3' )** |  |
| --- | --- | --- | --- |
| *ADAM9* | F* | GAACTGTGGCGAGTGTCTGA |  |
|  | R* | AGGCTACTACATCCCAGGCA |  |
| *ADAM10* | F | ATGGTGAGCCCATTGACAGG |  |
|  | R | AAGGGAGCCTGATGAGGGAT |  |
| *ADAM12* | F | CTGGGCCACAATTTCGGGATGAAT |  |
|  | R | ACTGCTGAACACCATGGGAAATGG |  |
| *ADAM17* | F | GGTGAGATGCAGGCTCTTGT |  |
|  | R | ATTCCCTCCTCTCCCACCTC |  |
| *MICA* | F | ACCCTCCCTGTGCTATGGAT |  |
|  | R | TCCGGGGATAGAAGCTGGAA |  |
| *GAPDH* | F | GTCTCCTCTGACTTCAACAGCG |  |
|  | R | ACCACCCTGTTGCTGTAGCCAA |  |

* F and R represent forward and reverse primers, respectively, used for PCR amplification.


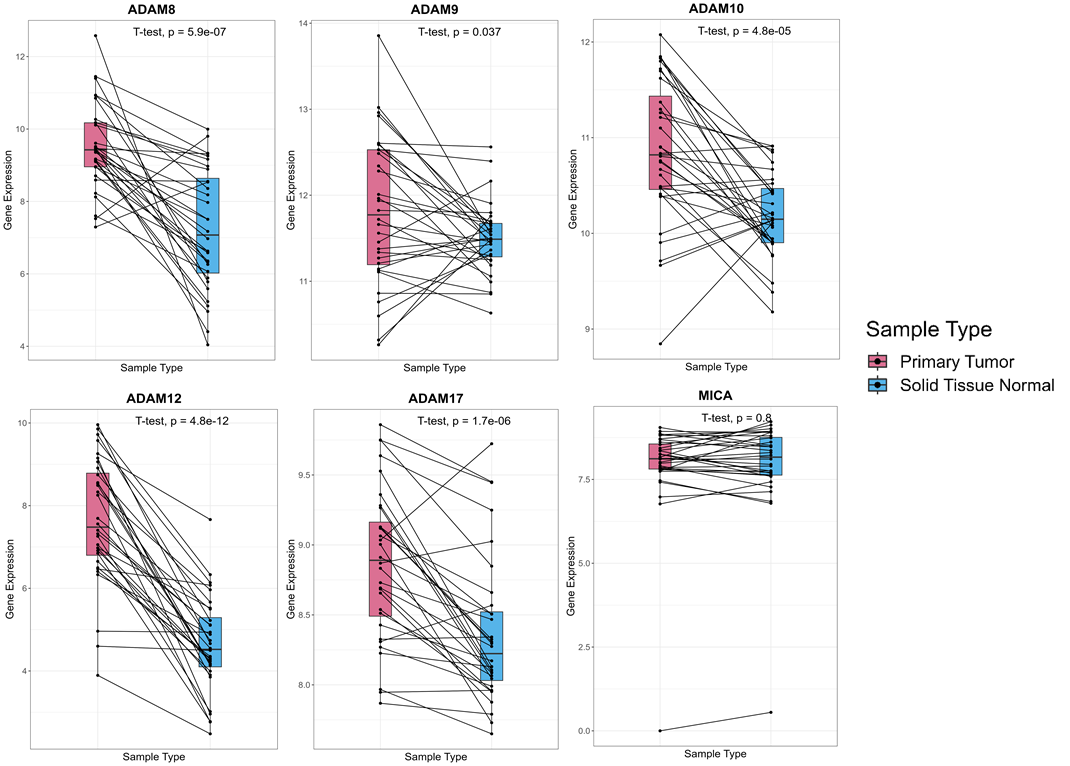


**Supplementary Figure S1. *ADAM*** **and *MICA* mRNA expression in gastric cancer tissues compared with paired normal tissues from the same patients in TCGA database.** Gastric cancer tissues (n = 32) were compared with the paired normal tissues (n = 32) derived from the same patients by paired t-test.


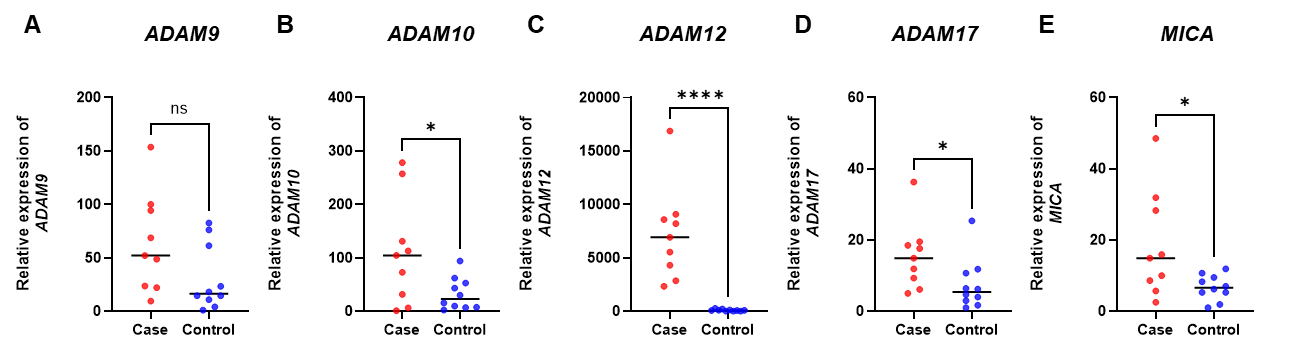


**Supplementary Figure S2. *ADAM* and *MICA* mRNA expression in the training set of plasma samples of early gastric cancer patients and healthy controls.** Expression levels of mRNAs for (A) *ADAM9* (*p* = 0.0789), (B) *ADAM10* (*p* = 0.031), (C) *ADAM12* (*p* < 0.0001), (D) *ADAM17* (*p* = 0.0279), and (E) *MICA* (*p* = 0.0334) were examined using qRT-PCR in plasma samples obtained from early gastric cancer patients (case, n=9, shown in red) and healthy controls (control, n=10, shown in blue). The data were normalized to an internal control (GAPDH) and presented as relative expression. * *p* < 0.05, ** *p* < 0.005, *** *p* < 0.001, **** *p* < 0.0001, ns. not specific; t-test or Mann-Whitney U test.


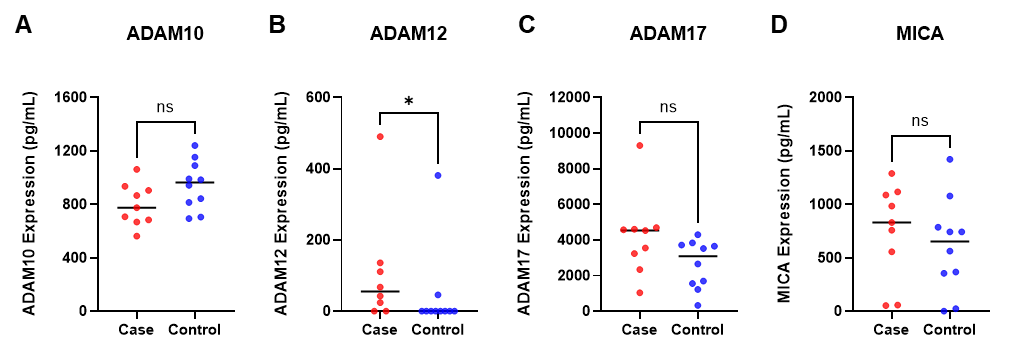


**Supplementary Figure S3. ADAM and MICA protein expression in the training set of serum samples of early gastric cancer patients and healthy controls.** Expression levels of (A) ADAM10 (*p* = 0.075), (B) ADAM12 (*p* = 0.039), (C) ADAM17 (*p* = 0.084), and (D) MICA (*p* = 0.504) proteins were examined using ELISA in serum samples obtained from early gastric cancer patients (case, n=9 [n=8 for ADAM12], shown in red) and healthy controls (control, n=10, shown in blue). * *p* < 0.05, ** *p* < 0.005, *** *p* < 0.001, **** *p* < 0.0001, ns. not specific; t-test or Mann-Whitney U test.


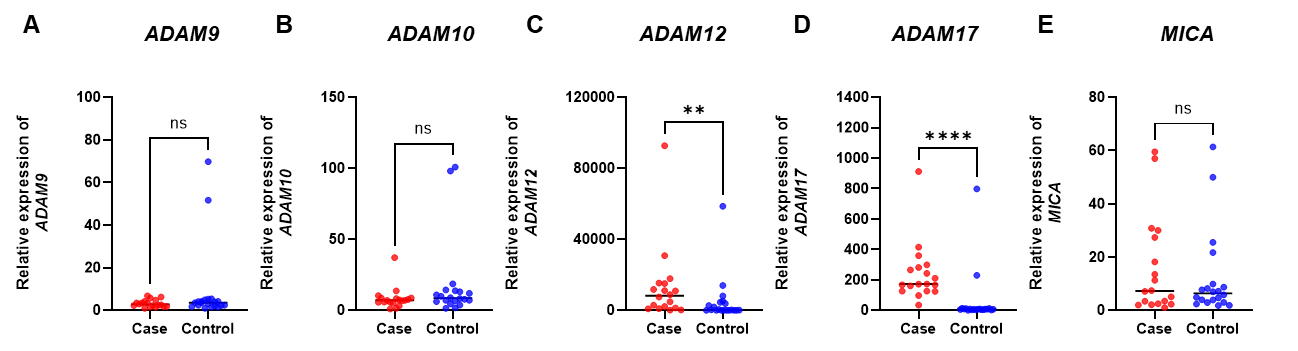


**Supplementary Figure S4. *ADAM* and *MICA* mRNA expression in the validation set of plasma samples of early gastric cancer patients and healthy controls.** Expression levels of mRNAs for (A) *ADAM9* (*p* = 0.519), (B) *ADAM10* (*p* = 0.135), (C) *ADAM12* (*p* = 0.0023), (D) *ADAM17* (*p* < 0.0001), and (E) *MICA* (*p* = 0.762) were examined using qRT-PCR in plasma samples obtained from early gastric cancer patients (case, n=18, shown in red) and healthy controls (control, n=20, shown in blue). The data were normalized to an internal control (GAPDH) and presented as relative expression. * *p* < 0.05, ** *p* < 0.005, *** *p* < 0.001, **** *p* < 0.0001, ns. not specific; t-test or Mann-Whitney U test.


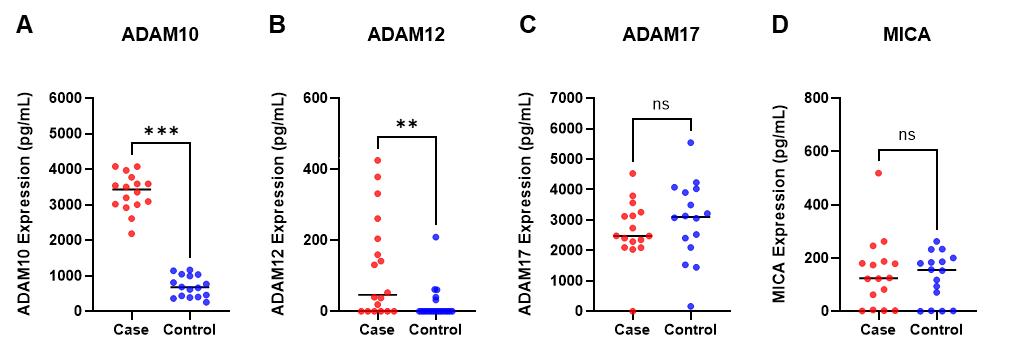


**Supplementary Figure S5. ADAM and MICA protein expression in the validation set of serum samples of early gastric cancer patients and healthy controls.** Expression levels of (A) ADAM10 (*p* < 0.001), (B) ADAM12 (*p* = 0.005), (C) ADAM17 (*p* = 0.403), and (D) MICA (*p* = 0.948) were examined using ELISA in serum samples obtained from early gastric cancer patients (case, n=16, shown in red) and healthy controls (control, n = 16 [n = 15 for ADAM12], shown in blue). * *p* < 0.05, ** *p* < 0.005, *** *p* < 0.001, **** *p* < 0.0001, ns. not specific; t-test or Mann-Whitney U test.


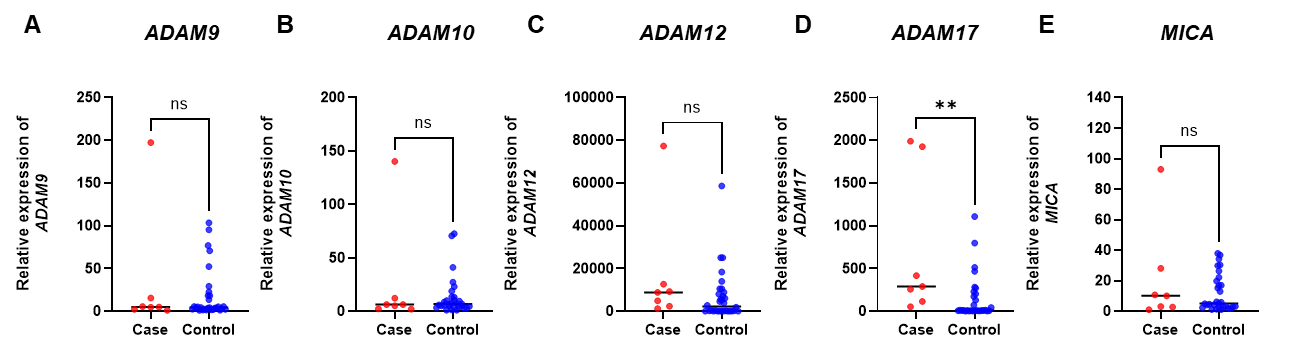


**Supplementary Figure S6. *ADAM* and *MICA* mRNA expression in the combined sets of plasma samples of advanced gastric cancer patients and healthy controls.** mRNA expression levels of (A) *ADAM9* (*p* = 0.887), (B) *ADAM10* (*p* = 0.306), (C) *ADAM12* (*p* = 0.133), (D) *ADAM17* (*p* = 0.003), and (E) *MICA* (*p* = 0.217) were examined using qPCR in plasma samples obtained from advanced gastric cancer patients (AGC, n=7, shown in red) and healthy controls (control, n=30, shown in blue). The data were normalized to an internal control (GAPDH) and presented as a relative expression. * *p* < 0.05, ** *p* < 0.005, *** *p* < 0.001, **** *p* < 0.0001, ns. not specific; t-test or Mann-Whitney U test.


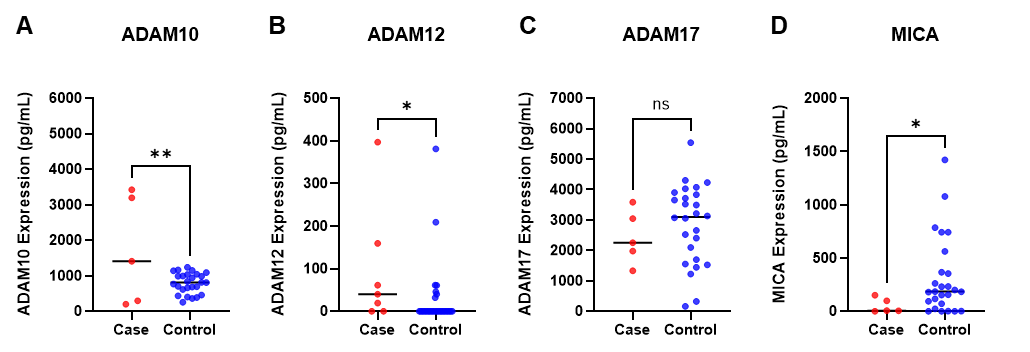


**Supplementary Figure S7. ADAM and MICA protein expression in the combined sets of serum samples of advanced gastric cancer patients and healthy controls.** Expression levels of (A) ADAM10 (*p* = 0.006), (B) ADAM12 (*p* = 0.014), (C) ADAM17 (*p* = 0.496), and (D) MICA (*p* = 0.042) were examined using ELISA in serum samples obtained from early gastric cancer patients (case, n=5, shown in red) and healthy controls (control, n=26 [n=25 for ADAM12], shown in blue). * *p* < 0.05, ** *p* < 0.005, *** *p* < 0.001, **** *p* < 0.0001, ns, not specific; t-test or Mann-Whitney U test.
